# Comparison of IL‐2‐antibody to IL‐2‐Fc with or without stereotactic radiation therapy in CEA immunocompetent mice with CEA positive tumors

**DOI:** 10.1002/cam4.6909

**Published:** 2024-02-05

**Authors:** Lindsay Williams, Lin Li, Paul J. Yazaki, Patty Wong, Teresa Hong, Erasmus K. Poku, Susanta Hui, Hemendra Ghimire, John E. Shively, Maciej Kujawski

**Affiliations:** ^1^ Department of Immunology and Theranostics, Riggs Diabetes, Metabolism, and Research Institute Beckman Research Institute of City of Hope Duarte California USA; ^2^ Radiopharmacy City of Hope Duarte California USA; ^3^ Beckman Research Institute of City of Hope Duarte California USA; ^4^ Department of Radiation Oncology City of Hope Duarte California USA; ^5^ City of Hope Medical Center Duarte California USA

**Keywords:** breast cancer, colon cancer, immunocytokines, immunotherapy, radiation therapy

## Abstract

**Background:**

The potent immune effects of interleukin‐2 (IL‐2) for cancer therapy can be increased by genetic fusion of IL‐2 to the Fc domain of an antibody (IL‐2‐Fc) or tumor targeted by genetic fusion to a whole antibody known as an immunocytokine (ICK).

**Methods:**

An anti‐CEA ICK (M5A‐IL‐2) was compared to an IL‐2‐Fc fusion protein using tumor therapy and PET imaging in CEA transgenic immunocompetent mice bearing CEA positive colon or breast tumors. Combination with stereotactic radiation therapy (SRT) was performed with either ICK or IL‐2‐Fc.

**Results:**

ICK and IL‐2‐Fc had comparable antitumor effects in both tumor models, although ICK had higher tumor uptake and slower blood clearance than an IL‐2‐Fc. Analysis of IFNγ^+^/CD8^+^ and FoxP3^+^/CD4^+^ T cells revealed higher levels of IFNγ‐producing CD8^+^ T cells in ICK treated mice versus more efficient Treg elimination in IL‐2‐Fc treated mice. No significant or lasting toxicity was detected for either agent. Combination therapies with SRT revealed comparable efficacy and induction of immune memory for both ICK and IL‐2‐Fc when mice were rechallenged post‐therapy.

**Conclusions:**

IL‐2‐Fc had comparable antitumor efficacy to CEA‐targeted M5A‐IL‐2 ICK, while both fusion proteins induced immune memory when combined with SRT. Differences in the therapeutic mechanisms of both agents were observed.

## Graphical abstract

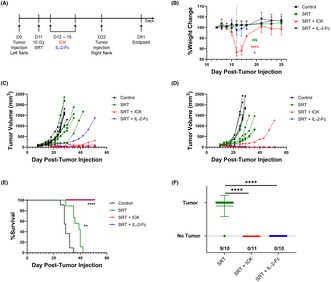

## 1 INTRODUCTION

Interleukin‐2 (IL‐2) is a T‐cell growth factor produced by activated CD4^+^ T cells through T‐cell receptor and CD28 complex signaling.^1^, ^2^ IL‐2 can propagate an inflammatory immune response but can also play an inhibitory role via binding to interleukin 2 receptor α‐chain (IL‐2Rα; CD25) highly expressed on regulatory T cells (Tregs).^3^ Most IL‐2 based antitumor therapy designs work to decrease Treg‐mediated immunosuppression and tolerance and/or enhance cytotoxic T cell activation. When administered alone, IL‐2 exhibits many therapeutic limitations such as a short serum half‐life and a requirement for high therapeutic doses with resultant toxicities.^4^ These toxicities include vascular leak syndrome (VLS), pulmonary edema, hypotension, and heart myopathy.^4^, ^5^, ^6^, ^7^ These toxicities are mediated by IL‐2‐induced production of IL‐1, IL‐6, TNFα, IFNγ, and IL‐5 and downstream effects.^4^, ^8^

To overcome various limitations of IL‐2 therapy for cancer, IL‐2 has been engineered into many formats that work to increase half‐life and minimize Treg activation. One common format is the fusion of IL‐2 and the Fc portion of an antibody. Most notably, the fusion of IL‐2 to Fc derived from antibody subtypes with high effector function (such as murine IgG2 or human IgG1) yields therapeutic agents with low toxicity and high antitumor activity mediated by Fc‐mediated Treg depletion and moderate systemic immune cell activation.^9^, ^10^ In addition, IL‐2 fusion proteins must also be screened for aggregation due to the natural propensity of IL‐2 to aggregate. To minimize aggregation commonly seen with IL‐2‐Fc, the K35E mutation in the IL‐2 moiety was incorporated in a fully human IL‐2 IgG1 Fc fusion protein construct.^11^ The resulting IL‐2‐Fc fusion protein was shown to have low toxicity and high antitumor efficacy in melanoma, colon cancer, and breast cancer models.^10^, ^11^ However, a number of aspects of IL‐2‐Fc tumor therapy have not been studied, including quantitative tumor targeting and comparison to other fusion proteins such as immunocytokines (ICKs) containing IL‐2.

ICKs employ the immune activating potential of cytokines fused to tumor targeting antibodies, leading to high antitumor efficacy and low toxicity.^12^ We previously developed a monoclonal antibody that targets carcinoembryonic antigen (CEA; CEACAM5) using clinically proven humanized CEA‐specific hT84.66‐M5A (M5A) for use in both imaging^13^, ^14^, ^15^ and therapy studies.^16^, ^17^ CEA is highly expressed in the majority of colon cancers, as well as many other solid tumors such as breast, ovarian, and pancreatic tumors.^18^, ^19^, ^20^, ^21^ We recently developed an M5A‐human IL‐2 fusion protein based on initial successes using a full murine version of our anti‐CEA antibody and murine IL‐2.^13^, ^22^, ^23^ M5A‐IL‐2 ICK displayed potent antitumor effects in CEA^+^ breast and colon cancers using an immunocompetent mouse model with human CEA transgene expression, including tumor eradication and induction of immune memory when combined with stereotactic radiation therapy (SRT).^13^

Based on previous data, we know that an IL‐2‐Fc (containing K35E in the human IL‐2 moiety; composed of human IL‐2 and human IgG1 Fc) and M5A‐IL‐2 ICK have minimal aggregation and maintain CD25 binding and IL‐2 activation.^10^, ^13^ We now compare IL‐2‐Fc to tumor‐targeted M5A‐IL‐2 ICK in CEA^+^ solid breast and colon cancer models in CEA transgenic mice. Early and late time points were assessed for changes in immune landscape and potential toxicities. Further studies were conducted between IL‐2‐Fc and M5A‐IL‐2 ICK combined with SRT to compare tumor size reduction and generation of immune memory. PET, biodistribution, toxicity, and pharmacokinetics analyses were also performed for each agent to determine what factors affected therapy in two murine tumor models.

## 2 MATERIALS AND METHODS

### 2.1 Materials

^64^CuCl_2_ (74 mBq/0.05 mL in 0.01 M HCl) was acquired from the Mallinckrodt Radiological Institute, Washington University, St. Louis, MO. IL‐2‐Fc (K35E and C125S in the human IL‐2 moiety, human IgG1) and M5A‐IL‐2 ICK were produced as described previously.^10^, ^11^, ^13^, ^23^ Treatment agents referred to as IL‐2‐Fc and ICK in results section.

### 2.2 Cell culture

The E0771/CEA breast and MC38/CEA colon cancer cell lines were produced via stable transfection with a plasmid containing CEA as previously described.^22^, ^24^ Cell lines were cultured in DMEM (4.5 g/L glucose; Corning™) supplemented with 10% heat inactivated fetal bovine serum (FBS; Corning™), 1 × Antibiotic Antimycotic Solution (Corning™), and 1 × GlutaMAX™ (gibco™). Cell cultures were tested for mycoplasma quarterly using a mycoplasma detection kit (Lonza MycoAlert™ Mycoplasma Detection Kit).

### 2.3 Animal studies

In vivo mouse experiments and mouse care were performed under pathogen‐free conditions per established institutional guidance and approved protocols from the Institutional Animal Care and Use Committee of Beckman Research Institute of City of Hope National Medical Center (IACUC#: 91037). CEA transgenic (CEAtg) mice were used for all animal experiments as previously described.^13^, ^24^ Since the CEA gene (also known as CEACAM5) is not present in mice, these mice were chosen to prevent immunological rejection of CEA as a foreign gene.^24^ These CEAtg mice are on a C57BL/6 background and have equivalent CEA expression in tissues similar to that seen in humans (ie., gastrointestinal organs).^24^ In the E0771/CEA breast carcinoma model, 1 × 10^5^ tumor cells in PBS:matrigel 1:1 ratio (50 μL) were injected orthotopically into the mammary fat pad. For the colon cancer model, 1 × 10^6^ MC38/CEA tumor cells (total volume 50 μL) were injected subcutaneously. To test establishment of immune memory in colon cancer model, some of the mice received a second subcutaneous injection of MC38/CEA cells on the opposite flank. Four daily M5A‐IL‐2 ICK (1 mg/kg) and molar equivalent doses of IL‐2‐Fc were delivered for each dose. Short term analyses were performed 1 day posttreatment. In the SRT combination study, tumor‐bearing mice were treated with the Precision X‐RAD SMART Plus/225cx (Precision x‐Ray, North Branford, CT) as performed previously.^13^ Treatment groups were given a single dose of 10 Gy per tumor. Mice were euthanized if a tumor exceeded 1500 mm^3^.

### 2.4 PET imaging studies

IL‐2‐Fc (1.5 mg/mL) in PBS was conjugated to DOTA‐NHS‐ester (Macrocyclics, B280) at a 20:1 molar ratio of DOTA‐NHS‐ester to protein. M5A‐IL‐2 ICK was conjugated at a 15:1 molar ratio of DOTA‐NHS‐ester to protein. After 2 h at RT on a rotator, samples were dialyzed versus PBS (5 × 5 L) and sterile filtered. Conjugates were verified by IEF gel electrophoresis (data not shown). Immunoreactivity of ^64^Cu radiolabeled samples to CEA and CD25‐Fc was confirmed by addition of excess CEA or CD25‐Fc and run on size exclusion chromatography (data not shown). PET imaging studies were performed in CEAtg mice bearing E0771/CEA tumors as previously described.^13^, ^14^ PET scans were acquired using a GNEXT microPET/CT scanner (SOFIE). Mice were injected with a single intravenous dose of 100 μCi (10 μg) of each ^64^Cu‐DOTA‐labeled product in 1% human serum albumin–buffered saline through a tail vein catheter.^13^ At 0, 4, 24, and 48 h, cheek blood samples were collected from mice injected with ^64^Cu‐DOTA‐IL‐2‐Fc or ^64^Cu‐DOTA‐M5A‐IL‐2 ICK for pharmacokinetic (PK) analysis. At the terminal PET time point (24 h), the mice were euthanized and biodistribution studies were performed.

### 2.5 Leukocyte analysis

Tumors, tumor draining lymph nodes (TDLNs), spleens, and blood were collected at each mouse study endpoint. Tissues and blood were then processed and assessed via flow cytometry (BD LSRFortessa™ Cell Analyzer) using a live cell gating strategy for different immune cell subsets as described previously (raw data available upon request).^13^, ^25^ Different combinations of fluorochrome‐labeled antibodies to murine CD3, CD4, CD8, B220, CD19, CD11b, Ly6C, Ly6G, CD11c, F4/80, and PD‐1 (BioLegend®) were used to stain cell suspensions. Tumor, spleen, and TDLN T cell were analyzed for IFNγ production and FoxP3 expression by intracellular flow cytometry as described previously.^13^ In some studies, myeloid tumor subpopulations were analyzed for iNOS expression by intracellular flow cytometry.

### 2.6 Lung tissue H&E

Lung tissues were harvested and immediately fixed in 10% neutral buffered formalin. Dehydration, clear, and paraffinization were performed on a Tissue‐Tek VIP Vacuum Infiltration Processor (SAKURA). The samples were embedded in paraffin using a Tissue‐Tek TEC Tissue Embedding Station (SAKURA). Samples were then sectioned at 5 μm and put on positively charged glass slides. The slides were deparaffinized, rehydrated, and stained with Modified Mayer's Hematoxylin and Eosin Y (H&E) Stain (America MasterTech Scientific) on an H&E Auto Stainer (Prisma Plus Auto Stainer, SAKURA) according to standard laboratory procedures. The stains were visualized with DISCOVERY ChromoMap DAB Kit, counterstained with hematoxylin (Ventana) and cover‐slipped. H&E stained slides were digitalized and documented by iScan HT (Roche) scanner, and then representative pictures (10× magnified) were taken from the NDP.view2 viewing software.

### 2.7 Cytokine analysis

Plasma was assayed for murine IFNγ, IL‐5, IL‐10, IL‐2, and TNFα using ELISA kits (BD OptEIA™) and uncoated Nunc™ MaxiSorp™ ELISA Plates (BioLegend®) according to the manufacturer's protocol and analyzed using the SpectraMax M2 (Molecular Devices) plate reader.

### 2.8 Statistical analysis

GraphPad Prism 8 software was used to generate all graphs and statistical significance (grouped unpaired *t*‐tests). FlowJo™ software was used to analyze flow cytometry data and generate gated percentages used in GraphPad Prism 8 graphs. Adobe Photoshop 2020 was used to create all figure files.

## 3 RESULTS

### 3.1 IL‐2‐Fc has comparable efficacy to M5A‐IL2 ICK in an MC38/CEA colon cancer mouse model

**FIGURE 1:**
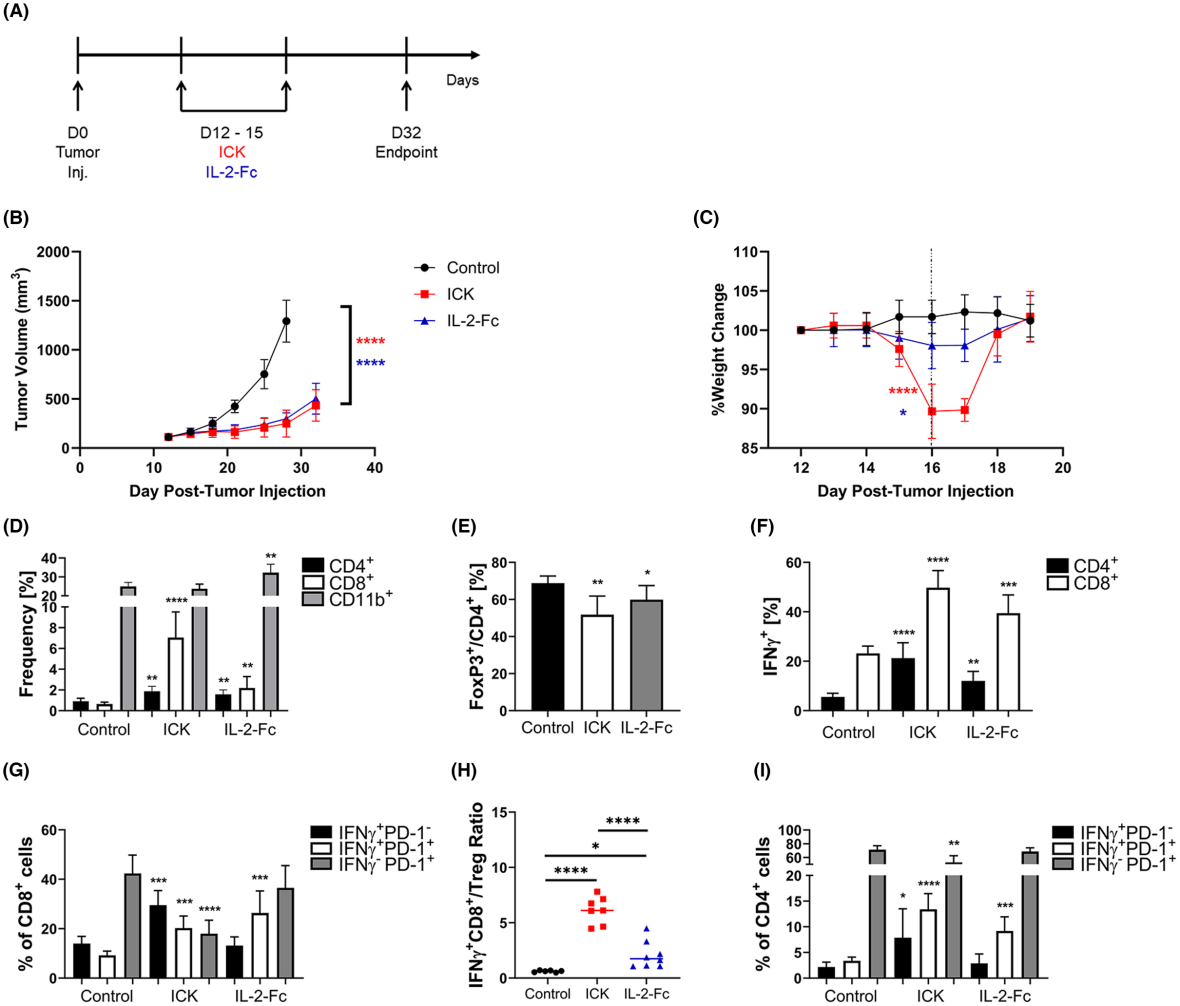
ICK and IL‐2‐Fc therapy in MC38/CEA subcutaneous colon tumors. (A) Study design scheme. Subcutaneous MC38/CEA (1 × 10^6^) tumor‐bearing CEAtg mice were treated on Days 12–15 after tumor implantation with intraperitoneal injection of 1 mg/kg ICK or molar equivalent of IL‐2‐Fc and euthanized on Day 32. (B) Tumor growth curves of control (untreated), ICK, and IL‐2‐Fc treatment groups (*n* = 6–8 per group). (C) Percent weight change from baseline (100%). Dashed line represents time point chosen for statistical comparison (*n* = 7–8 per group). (D–I) Flow analysis of cell frequencies from endpoint tumor digests (*n* = 6–8 per group). (D) CD4^+^, CD8^+^, and CD11b^+^ leukocytes. (E) FoxP3^+^/CD4^+^ (Tregs). (F) IFNγ^+^ CD4^+^ and CD8^+^ T cells. (G) IFNγ^+^PD‐1^−^, IFNγ^+^PD‐1^+^, and IFNγ^−^PD‐1^+^ CD8^+^ T cells. (H) IFNγ^+^CD8^+^/Treg ratio (out of all live cells in tumor). (I) IFNγ^+^PD‐1^−^, IFNγ^+^PD‐1^+^, and IFNγ^−^PD‐1^+^ CD4^+^ T cells. ****p < 0.0001; ***p < 0.001; **p < 0.01; *p < 0.05. (All statistics shown are in comparison to control values unless explicitly shown).

The effects of targeted M5A‐IL‐2 ICK (ICK) versus untargeted IL‐2‐Fc were compared in a subcutaneous MC38/CEA colon cancer model. ICK and IL‐2‐Fc monotherapies were administered in equimolar amounts over 4 consecutive days in CEA transgenic (CEAtg) mice with established subcutaneous solid tumors expressing CEA (Figure 1A). The tumor growth curves for each therapy (Figure 1B) showed equivalent efficacy between ICK and IL‐2‐Fc treatments. As a measure of systemic toxicity, whole body weight loss was monitored (Figure 1C). ICK caused greater temporary weight loss than IL‐2‐Fc, although both treatments exhibited the same recovery kinetics within a few days posttreatment (see Day 18 weights in Figure 1C).

Tumors from treated groups showed consistently elevated CD4^+^ and CD8^+^ T cells, decreased Tregs, and increased T cell IFNγ production (Figure 1D–F). However, ICK treated tumors showed a significantly higher increase in CD8^+^ T cells compared to IL‐2‐Fc treated tumors, whereas IL‐2‐Fc treated tumors had significantly higher CD11b^+^ cells than untreated control or ICK groups (Figure 1D). Each therapy caused significant increases in IFNγ^+^PD‐1^+^ CD8^+^ T cells (Figure 1G). However, the proportions of IFNγ^+^PD‐1^−^ and of IFNγ^−^PD‐1^+^ CD8^+^ T cells between treated groups differed, with ICK treated groups showing significant increases in IFNγ^+^PD‐1^−^ and decreases in IFNγ^−^PD‐1^+^ CD8^+^ T cells not seen in IL‐2‐Fc treated tumors (Figure 1G). Interestingly, only ICK caused a significant decrease in IFNγ^+^PD‐1^+^TIM3^+^ CD8^+^ T cells (Figure S1A), suggesting CD8^+^ T cells in ICK‐treated tumors exhibited less exhaustion than CD8^+^ T cells in control or IL‐2‐Fc‐treated tumors. In terms of immune activation versus suppression, ICK caused a significantly higher IFNγ^+^CD8^+^/Treg T cell ratio compared to control or IL‐2‐Fc groups, whereas IL‐2‐Fc treatment resulted in a moderate (*p* = 0.0128) increase in the IFNγ^+^CD8^+^/Treg ratio (Figure 1H). These results demonstrate an overall increase in cytotoxic CD8^+^ T cells and decrease in immunosuppressive Tregs, with ICK treatment having a larger impact on immune cell composition in the tumor. Regarding other CD4^+^ T cell phenotypes, ICK treated tumors showed significant increases in IFNγ^+^PD‐1^−^ and IFNγ^+^PD‐1^+^ and a decrease in IFNγ^−^PD‐1^+^ CD4^+^ T cells, whereas IL‐2‐Fc treated tumors only showed a significant increase in IFNγ^+^PD‐1^+^ CD4^+^ T cells (Figure 1I). Overall, changes in PD‐1^+^ populations were similar for CD8^+^ and CD4^+^ T cells with both therapies.

Endpoint spleen masses were marginally lower for ICK compared to IL‐2‐Fc therapy or controls (Figure S1B). Endpoint spleen and TDLN immune cell composition were also analyzed for each experimental group to determine the extent of extra‐tumoral immune composition changes. Only IL‐2‐Fc showed a significant decrease in splenic Tregs compared to ICK and control groups (Figure S1C). However, neither ICK nor IL‐2‐Fc showed a significant decrease in TDLN Tregs (Figure S1D). ICK and IL‐2‐Fc therapies both caused significant increases in splenic CD8^+^ T cells expressing IFNγ, whereas only ICK caused a significant increase in splenic CD4^+^ T cells expressing IFNγ (Figure S1E). TDLN T cell IFNγ expression had a similar pattern as that seen in the spleen with each treatment (seen in Figure S1F). This suggests persistent T‐cell activation not only in tumors but also in the periphery. Additionally, endpoint lung sections were H&E stained to assess lung toxicity with ICK or IL‐2‐Fc treatment. These lung sections showed no evidence of increased cellularity with ICK or IL‐2‐Fc compared to controls (Figure S2A–C), suggesting that although both treatments caused transient weight loss, there was no evidence of lung damage.

The MC38/CEA study results suggest equivalent but distinct therapeutic mechanisms between ICK and IL‐2‐Fc. ICK causes a more significant increase in the IFNγ^+^CD8^+^/Treg ratio compared to IL‐2‐Fc, and IL‐2‐Fc also appears to have fewer toxic effects than ICK based on temporary weight loss.

### 3.2 IL‐2‐Fc and ICK have comparable antitumor effects in an E0771/CEA orthotopic breast cancer model

To compare efficacies in a different tumor model, orthotopic E0771/CEA breast tumors in CEAtg mice were treated with equimolar amounts of IL‐2‐Fc and ICK over 4 consecutive days (Figure S3A). Tumor growth curves for IL‐2‐Fc versus ICK therapy showed similar tumor growth inhibition (Figure S3B). Each therapy also caused temporary total body weight loss, although IL‐2‐Fc caused the least weight loss (Figure S3C). Interestingly, female mice in this model took less time to recover their weight than male mice used in the colon cancer model (seen in Figure 1C) when treated with ICK.

Analysis of endpoint tumors (Day 21) from treated groups showed consistent reduction of Tregs and increased IFNγ production by CD8^+^ and CD4^+^ T cells (Figure S3D–F). ICK treatment caused a significant increase in IFNγ^+^PD‐1^+^ CD8^+^ T cells and decrease in IFNγ^−^PD‐1^+^ CD8^+^ T cells compared to IL‐2‐Fc and control, suggesting CD8^+^ T cells are more activated with ICK therapy (Figure S3G). In terms of immune activation versus regulation, the intratumoral IFNγ^+^CD8^+^/Treg ratio was not significantly elevated with IL‐2‐Fc treatment compared to a significant increase seen with ICK treatment (Figure S3H). This demonstrates ICK causes an overall increase in cytotoxic CD8^+^ T cells and/or decrease in Tregs, thereby eliciting a therapeutic stronger T‐cell based antitumor response (Figure S3H). Both ICK and IL‐2‐Fc do; however, show significantly increased IFNγ^+^ and decreased IFNγ^−^PD‐1^+^ CD4^+^ T cells compared to control tumors, suggesting ICK and IL‐2‐Fc mechanistically share induction of CD4^+^ T cell activation (Figure S3I).

Endpoint spleens (Day 21) were also assessed for weight differences and T cell composition (Figure S3J–L). Neither treatment caused a significant change in spleen weight in comparison to controls, although the proportions of Tregs, IFNγ^+^ CD8^+^ T cells, and IFNγ^+^ CD4^+^ T cells differed between treatments. IL‐2‐Fc treated mice had significantly lower splenic Tregs than ICK treated mice, suggesting IL‐2‐Fc has a slightly more Treg‐depleting effect in the periphery than ICK despite neither being significantly different from controls in this model at this time point (Figure S3K). ICK and IL‐2‐Fc therapies did cause significant increases in splenic CD8^+^ T cells expressing IFNγ, whereas only ICK caused a significant increase in splenic CD4^+^ T cells expressing IFNγ like what was seen in the MC38/CEA model (compare Figures S1D and S3L). The spleen analysis here suggests that IL‐2‐Fc has a more prolonged impact on Treg depletion, whereas ICK has a higher long‐term effect on increasing IFNγ^+^ CD8^+^ T cells in the periphery.

Despite contrasting therapeutic mechanisms between ICK and IL‐2‐Fc and small differences between mouse tumor models, in each instance ICK and IL‐2‐Fc treatments resulted in similar tumor growth inhibition due in large part to increased IFNγ^+^CD8^+^ T cells and/or decreased Tregs in the tumor.

### 3.3 Comparison of toxicities between ICK and IL‐2‐Fc in subcutaneous MC38/CEA colon cancer and orthotopic E0771/CEA breast cancer mouse models

**FIGURE 2:**
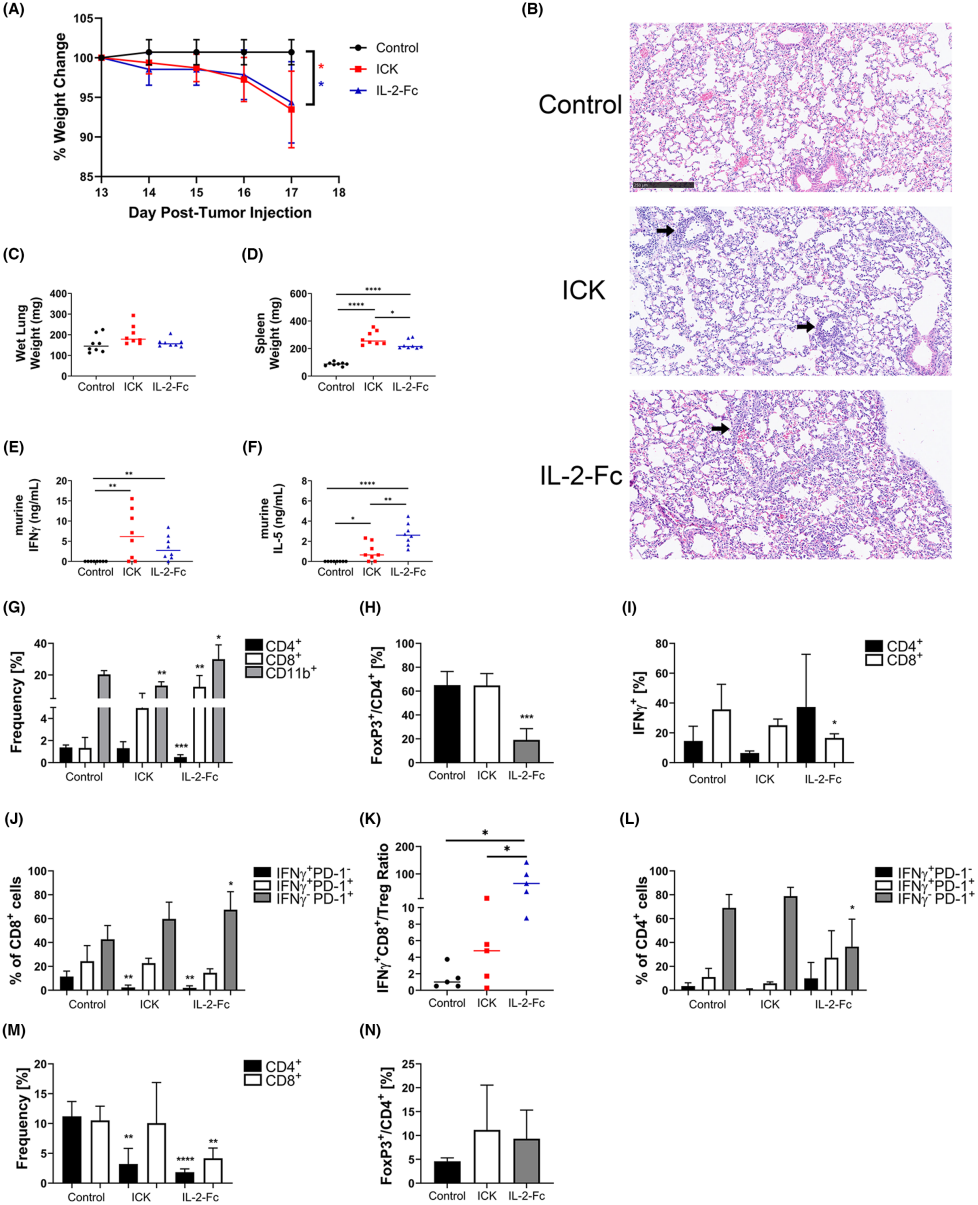
Comparison of ICK and IL‐2‐Fc toxicity in subcutaneous MC38/CEA mouse model. Mice processed 1 day posttreatment cessation following scheme in Figure 1A with treatment Days 13–16. *n* = 5–8 per group for all graphs shown. (A) Percent weight change from baseline (100%). (B) Representative H&E staining of endpoint lung section from control (untreated), ICK, and IL‐2‐Fc experimental groups (scale bar = 250 μm; all images at 10× magnification; black arrow indicates areas of increased cellularity near blood vessels). (C) Wet lung weight at endpoint. (D) Spleen weight at endpoint. (E) Plasma IFNγ concentration at endpoint. (F) Plasma IL‐5 concentration at endpoint. (G–N) Flow analysis of cell frequencies from endpoint samples. (G) CD4^+^, CD8^+^, and CD11b^+^ leukocytes in the tumor. (H) FoxP3^+^/CD4^+^ (Treg) cells in the tumor. (I) IFNγ^+^ CD4^+^ and CD8^+^ T cells in the tumor. (J) IFNγ^+^PD‐1^−^, IFNγ^+^PD‐1^+^, and IFNγ^−^PD‐1^+^ CD8^+^ T cells in the tumor. (K) IFNγ^+^CD8^+^/Treg ratio of all live cells in tumor. (L) IFNγ^+^PD‐1^−^, IFNγ^+^PD‐1^+^, and IFNγ^−^PD‐1^+^ CD4^+^ T cells in the tumor. (M) CD4^+^ and CD8^+^ T cells in the blood. (N) FoxP3^+^/CD4^+^ (Treg) cells in the blood. (*n* = 5 per group). ****p < 0.0001; ***p < 0.001; **p < 0.01; *p < 0.05. (All statistics shown are in comparison to control values unless explicitly shown).

To further explore the potential toxicity of ICK compared to IL‐2‐Fc shortly after the last (4th) therapeutic dose, additional studies were performed in both male and female CEAtg mice using the MC38/CEA and E0771/CEA mouse models, respectively. In each model, tissues were collected and analyzed 1 day posttreatment cessation following the schemes in Figure 1A; Figure S3A. Whole body toxicity, measured by weight change, was recorded for both models (MC38/CEA and E0771/CEA results shown in Figure 2A; Figure S4A, respectively), which indicated that the MC38/CEA tumor bearing male mice are more sensitive to IL‐2‐Fc treatment than E0771/CEA tumor bearing female mice due to higher weight loss seen in Figure 2A. Both models showed similar weight sensitivity with ICK treatment (Figure 2A; Figure S4A). All weight decreases were ~10% or less and recovered within 5 days, indicating minimal and transient toxicity in both models.

Lung sections were also stained to confirm if each treatment caused increased cellularity at this shorter time point posttreatment (Figure 2B; Figure S4B). Higher cellularity was observed with ICK treatment, although this effect was not seen in the MC38/CEA long term endpoint analysis, suggesting only transient lung toxicity (compare Figure 2B; Figure S2B). Gross lung toxicity was assessed by wet lung weight and peripheral immune cell expansion was assessed by spleen weight, seen in Figure 2C,D; Figure S4C,D. There were no significant differences in wet lung weight observed between experimental groups, although both models showed splenomegaly for ICK and IL‐2‐Fc treatment groups. This suggests that the increased cellularity observed in lung tissue staining was mild enough to not correlate with any observable wet lung weight changes, even though peripheral immune cell activation and expansion were occurring in these mice based on spleen enlargement (Figure 2B–D; Figure S4B–D). This contrasts with spleen weights returning close to control levels by the long‐term time point, further suggesting only transient potential toxicity is occurring with each therapy (Figures S1A and S3J). Plasma was also assessed for various cytokines, showing significant increases in circulating IFNγ, and IL‐5 in both models with ICK and IL‐2‐Fc treatments (Figure 2E,F; Figure S4E,F). Interestingly, the highest concentrations of IL‐5 were detected in mice treated with IL‐2‐Fc even though ICK caused slightly higher lung cellularity. Additionally, negligible concentrations of murine IL‐2, IL‐10, or TNFα were detected for either treatment group (data not shown).

To compare short term versus long term results, flow analysis of tumors at these early time points was performed and is summarized in Figure 2G–L; Figure S4G–L. Notably, IL‐2‐Fc consistently caused the most significant decrease in tumor Tregs compared to ICK at this earlier time point, which translated into IL‐2‐Fc's significantly higher IFNγ^+^CD8^+^/Treg ratio in both models compared to controls and ICK (Figure 2H,K; Figure S4H,K). This contrasts with observations shown in the long‐term therapy experiments presented above (Figure 2H; Figure S3H) where ICK treated mice had the highest average IFNγ^+^CD8^+^/Treg ratio. This suggests that IL‐2‐Fc treatment causes a stronger initial antitumor immune response than ICK due to far better elimination of Tregs, but ICK appears to maintain this response better in the long term mainly by increased IFNγ^+^CD8^+^ T cell tumor infiltration. Surprisingly, both ICK and IL‐2‐Fc caused significant increases in IFNγ^+^PD‐1^+^ CD4^+^ T cells specifically in the E0771/CEA tumors and significant decreases in IFNγ^+^PD‐1^−^ CD8^+^ T cells in the MC38/CEA tumors, suggesting model‐specific therapeutic mechanisms shared by both therapies at this early time point (Figure S4L; Figure 2J). Blood was also analyzed by flow cytometry to complement plasma cytokine and intratumoral analyses discussed above (Figure 2M,N; Figure S4M,N). IL‐2‐Fc and ICK caused significant decreases in circulating CD4^+^ T cells in both models (Figure 2M; Figure S4M), although no significant changes in circulating Tregs were observed in both models (Figure 2N; Figure S4N), suggesting changes in circulating T cell frequencies do not correlate with changes seen within the tumor.

Together, the early and late time point results from both models demonstrate only transient toxicity from ICK and IL‐2‐Fc monotherapies, emphasized by the body weight recovery by the study endpoint (Figure 1C; Figure S3C) as well as lung tissue recovery (compare Figure S2A–C; Figure 2B). The results also suggested shared and contrasting potential therapeutic mechanisms at play early in the course of these models despite the same endpoint phenotype of tumor growth inhibition.

### 3.4 Effects on myeloid cell subpopulations and iNOS expression between ICK and IL‐2‐Fc treatments in MC38/CEA and E0771/CEA tumor models

**FIGURE 3:**
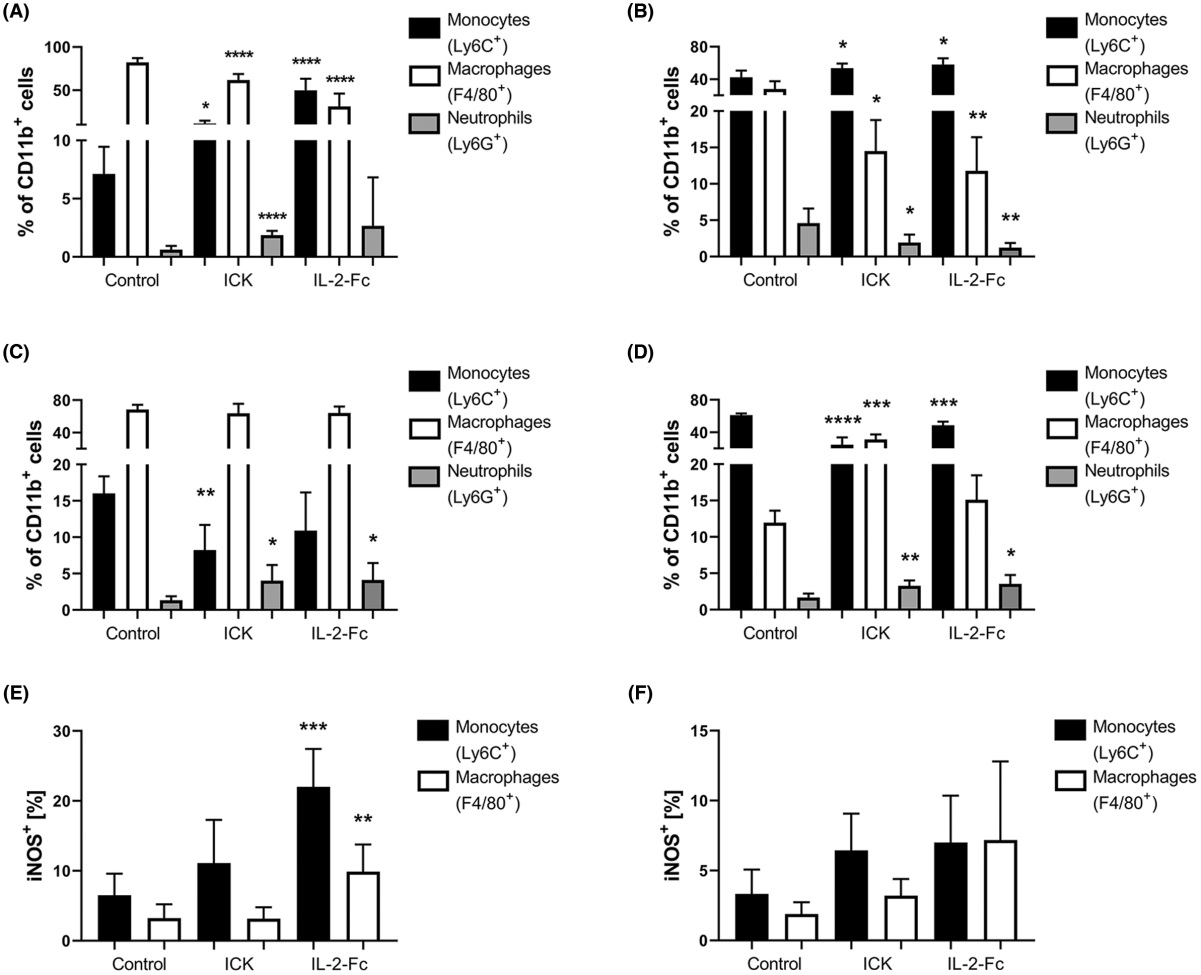
Intratumoral myeloid population analysis in MC38/CEA and E0771/CEA tumor models. (A–D) Monocytes (Ly6C^+^), macrophages (F4/80^+^), and neutrophils (Ly6G^+^) out of total myeloid (CD11b^+^) population in MC38/CEA (A and C) or E0771/CEA (B and D) tumors (*n* = 4–8 per group). (A) MC38/CEA tumor analysis on Day 32. (B) E0771/CEA tumor analysis on Day 21. (C) MC38/CEA tumor analysis on Day 17 (1 day posttreatment cessation). (D) E0771/CEA tumor analysis on Day 13 (1 day posttreatment cessation). (E) iNOS analysis from tumors in (C). (F) iNOS analysis from tumors in (D). ****p < 0.0001; ***p < 0.001; **p < 0.01; *p < 0.05; not significant (ns). (All statistics shown are in comparison to control values).

Tumor myeloid subpopulations were analyzed from the studies above to further explore the different mechanisms underlying the antitumor effects of ICK and IL‐2‐Fc (Figure 3). In the flow analysis of endpoint (Day 32) MC38/CEA tumors, ICK treatment showed a moderate (*p* = 0.0219) increase in CD11b^+^/Ly6C^+^ monocytes, whereas IL‐2‐Fc treatment resulted in a very significant (*p* < 0.0001) increase in potentially pro‐inflammatory CD11b^+^/Ly6C^+^ monocytes (Figure 3A). In comparison, endpoint (Day 21) E0771/CEA tumors with IL‐2‐Fc or ICK therapies exhibited similar moderate increases in CD11b^+^/Ly6C^+^ monocytes (Figure 3B). Both ICK and IL‐2‐Fc also shared significant reduction in CD11b^+^/F4/80^+^ macrophages in both models at these longer‐term time points (Figure 3A,B). Opposite trends were observed with regards to changes in CD11b^+^/Ly6G^+^ neutrophil proportions. For example, ICK caused a significant increase in intratumoral neutrophils in the MC38/CEA model, whereas ICK and IL‐2‐Fc caused a significant decrease in E0771/CEA intratumoral neutrophils (Figure 3A,B). This result implies both model‐specific differences in baseline neutrophil proportions as well as model‐specific responses to ICK and IL‐2‐Fc therapies.

Interestingly, monocyte frequencies significantly decreased with therapy in both models 1 day posttreatment despite significantly increasing at the longer time point (compare Figure 3C,D with Figure 3A,B). This suggests that there may be a recovery mechanism at play in the tumor after an immediate initial treatment response. Regarding macrophages, only ICK caused a significant increase 1 day posttreatment despite showing significant decreases at the longer‐term time point in E0771/CEA tumors only, suggesting a model and ICK‐specific therapeutic mechanism (compare Figure 3D with Figure 3B). Both therapies caused significant increases in neutrophils 1 day post‐therapy in both tumor models (Figure 3C,D). However, a significant decrease in neutrophils at the longer‐term time point was observed only for the ICK and IL‐2‐Fc treated E0771/CEA tumors, suggesting an E0771/CEA specific recovery mechanism posttreatment (compare Figure 3C,D with Figure 3A,B). Overall, the long‐ and short‐term results from Figure 3A–D suggest that ICK and IL‐2‐Fc show similar trends in myeloid subpopulation frequency changes, even though stronger shifts occur within different myeloid subsets with each therapy.

To further explore the tumor response at the short‐term time point, monocyte and macrophage inducible nitric oxide synthase (iNOS) expression was measured in both models using intracellular flow cytometry staining (Figure 3E,F). iNOS expression was chosen because it is a shared indicator of myeloid cell activation especially during a tumoricidal response.^26^ Although iNOS levels showed a trend toward increasing with each therapy, this increase was statistically significant only for IL‐2‐Fc treatment in the MC38/CEA model despite lack of correspondence to myeloid subpopulation changes (Compare Figure 3E with Figure 3C). Longer term iNOS expression is not shown because no significant shifts were observed (data not shown).

### 3.5 Stereotactic radiation therapy plus ICK or IL‐2‐Fc elicits immune memory in a MC38/CEA colon cancer model

**FIGURE 4:**
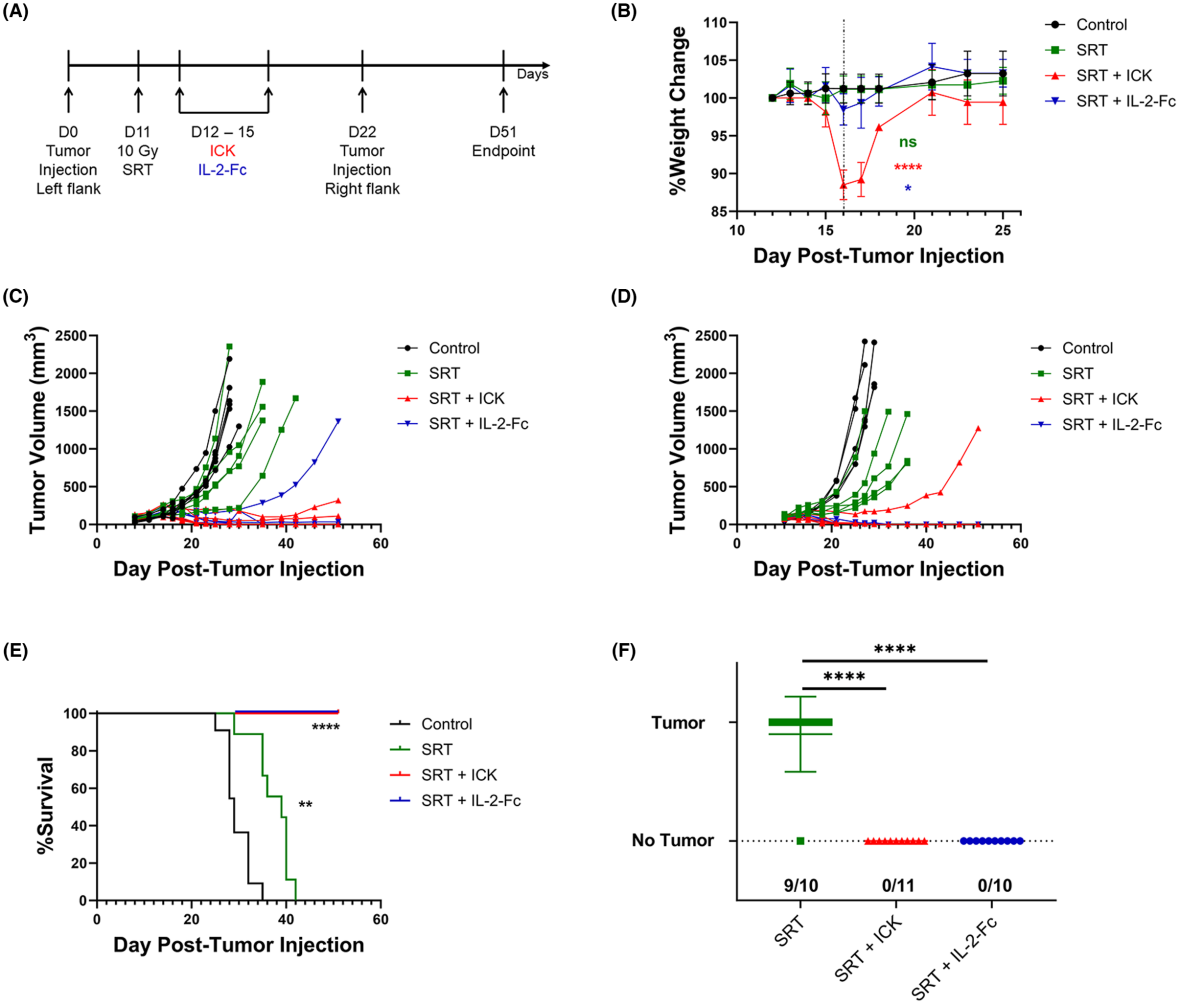
Generation of immune memory by stereotactic radiation therapy (SRT) + ICK or IL‐2‐Fc in subcutaneous MC38/CEA CEAtg mouse model. (A) Study design scheme. Subcutaneous MC38/CEA (1 × 10^6^) tumor‐bearing CEAtg mice were irradiated on Day 11 with 10 Gy SRT then treated on Days 12–15 post tumor implantation with intraperitoneal injection of 1 mg/kg recombinant ICK or molar equivalent of IL‐2‐Fc. The mice were rechallenged with subcutaneous MC38/CEA (1 × 10^6^) on the opposite (R) flank then euthanized on Day 51 post‐primary tumor injection. Mice were euthanized if a tumor exceeded 1500 mm^3^. (B) Percent weight change from baseline (100%). Dashed line represents time point chosen for statistical comparison. (one experiment of two shown; *n* = 5–6 per group). (C) Primary tumor growth curves of control (untreated), SRT, SRT + ICK, and SRT + IL‐2‐Fc experimental groups. (First experiment of two shown; *n* = 5–6 per group). (D) Primary tumor growth curves of control (untreated), SRT, SRT + ICK, and SRT + IL‐2‐Fc experimental groups. (Second experiment of two shown; *n* = 5 per group). (E) Kaplan–Meier plot of survival of mice from C and D. (Summary of two experiments, *n* = 10–11 per group). (F) Tumor outgrowth resulting from injection of secondary tumor on opposite, right flank (control mice not injected). (Summary of two experiments, *n* = 10–11 per group). ****p < 0.0001; **p < 0.01; *p < 0.05; not significant (ns).

Since we previously showed that SRT plus targeted ICK elicited immune memory in the MC38/CEA model,^13^ we combined SRT plus ICK or IL‐2‐Fc therapy to determine the added effect of tumor targeting in the same subcutaneous MC38/CEA mouse model. The therapy study scheme is depicted in Figure 4A and body weight changes are summarized in Figure 4B. The tumor growth curves for two study repeats with both agents plus SRT are shown in Figure 4C,D. In the majority of mice treated with SRT + ICK or SRT + IL‐2‐Fc (8/11 and 8/10 cases, respectively) primary tumors were completely eradicated by Day 51 (Figure 4C,D). Additionally, only the SRT + ICK combination therapy caused a 10% weight loss compared to 2% weight loss with SRT + IL‐2‐Fc, although all mice recovered within 5 days posttreatment (Figure 4B). The overall survival for all mice from Figure 4C,D is depicted in Figure 4E, showing that all of the SRT combination‐treated mice were still alive by the endpoint at Day 51. Mice were rechallenged with the same tumor cell injection parameters on the opposite flank at Day 22. Because of fast primary tumor growth in control animals, rechallenge injections were done only in therapeutic groups on mice from the experiments in Figure 4C,D. All but one of the rechallenge tumors grew in the SRT only group, whereas all rechallenge tumors in the combination therapy groups were rejected (Figure 4F). These results suggest that both ICK and IL‐2‐Fc can induce immune memory when combined with SRT, regardless of whether the primary tumor was completely eliminated.

### 3.6 PET, biodistribution, and pharmacokinetics of IL‐2‐Fc and M5A‐IL‐2 ICK in an orthotopic CEA transgenic mouse model

**FIGURE 5:**
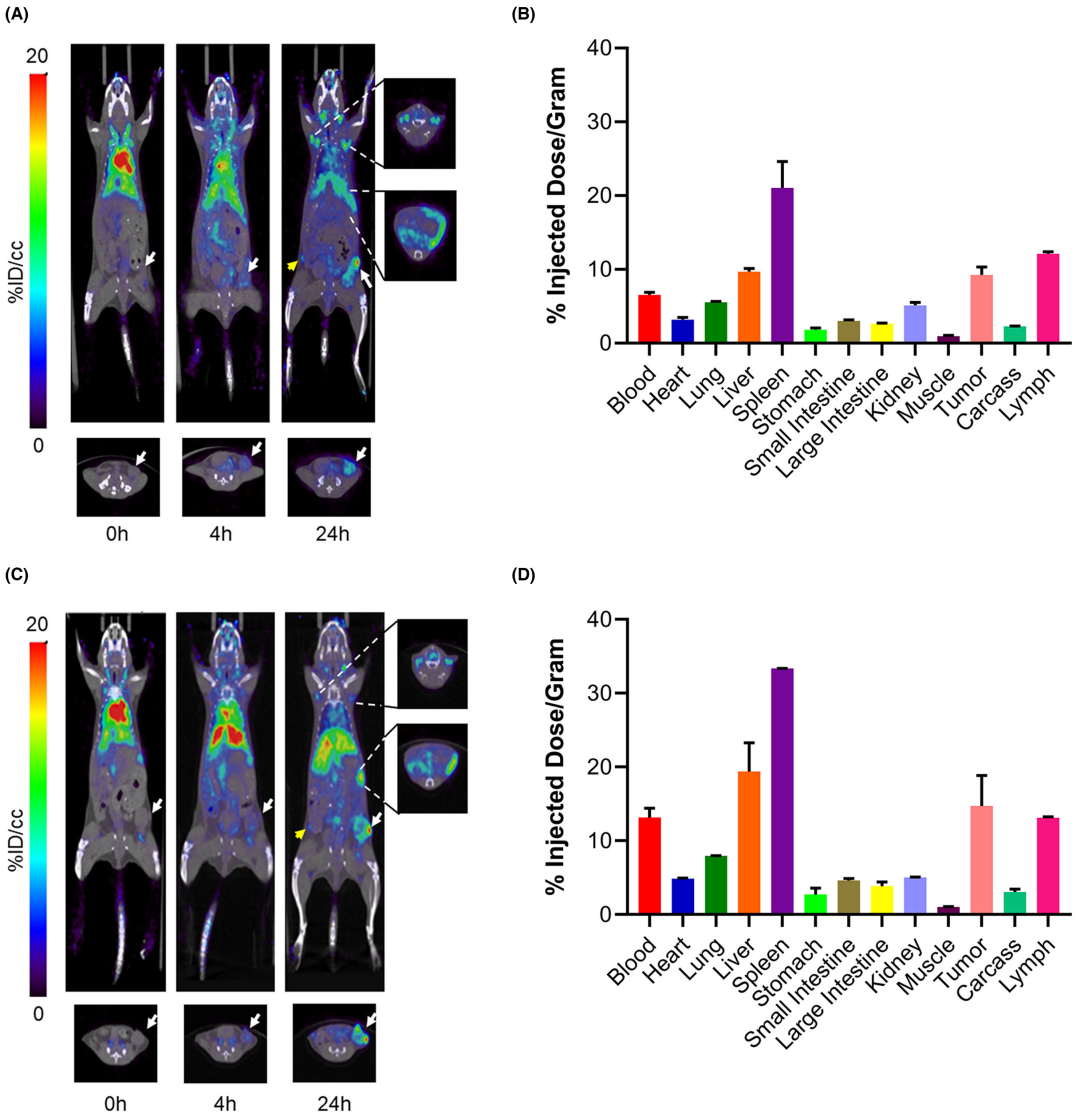
Tumor and immune organ targeting for IL‐2‐Fc and ICK. (A) Representative 0, 4, and 24 h transverse and coronal PET images of ^64^Cu‐DOTA‐IL‐2‐Fc in female CEA transgenic (CEAtg) mice with orthotopic E0771/CEA breast cancer (white arrow indicating tumor location; yellow arrow indicating inguinal lymph node opposite to tumor). (B) Biodistribution of material injected in (A) at 24 h postinjection. (C) Representative 0, 4, and 24 h transverse and coronal PET images of ^64^Cu‐DOTA‐ICK in female CEA transgenic mice with orthotopic E0771/CEA breast cancer (white arrow indicating tumor location; yellow arrow indicating inguinal lymph node opposite to tumor). (D) Biodistribution of material injected in (C) at 24 h postinjection. (*n* = 2 per group).

Tumor uptake, tissue biodistributions, and PK were compared between IL‐2‐Fc and ICK to determine their relative in vivo fates. IL‐2‐Fc and ICK were conjugated with NHS‐DOTA, radiolabeled with ^64^Cu, and injected into female CEA transgenic mice (CEAtg) bearing orthotopic E0771/CEA breast tumors. In the 24‐h PET images shown in Figure 5A, ^64^Cu‐DOTA‐IL‐2‐Fc shows immune organ uptake in lymph nodes (12.1 percent injected dose per gram (%ID/g)) and spleen (21.1 %ID/g), tumor uptake (9.3 %ID/g), and liver clearance (9.7 %ID/g). The 24‐h organ biodistribution for two mice is quantified in Figure 5B and shows a tumor to blood ratio of 1.4. For comparison, the PET imaging and biodistribution of ^64^Cu‐DOTA‐ICK shown in Figure 5C,D, respectively, shows an average of 14.7 %ID/g in the tumor with a tumor to blood ratio of 1.1. The ICK results show high immune organ and tumor uptake, but in contrast to ^64^Cu‐DOTA‐IL‐2‐Fc, the %ID/g of ^64^Cu‐DOTA‐ICK is higher in the blood (ICK: 13.1; IL‐2‐Fc: 6.5), liver (ICK: 19.4; IL‐2‐Fc: 9.7), spleen (ICK: 33.3; IL‐2‐Fc: 21.1), and tumor (ICK: 14.7; IL‐2‐Fc: 9.3) at 24 h postinjection. Blood clearance for radiolabeled IL‐2‐Fc and ICK from 0 to 48 h is shown in Figure S5A,B, respectively. The rate of blood clearance for ^64^Cu‐DOTA‐IL‐2‐Fc (54.7 %ID remaining in blood) was greater than that of ^64^Cu‐DOTA‐ICK (65.0 %ID remaining in blood) at 4 h postinjection, although at 24 h, both had near equivalent amounts (~20% of initial dose) remaining in the blood. When comparing these results to the therapeutic studies above, it appears that although more ICK goes to the tumor postinjection, both ICK and IL‐2‐Fc remain equally efficacious supported by strong immune organ uptake.

## 4 DISCUSSION

As a first in the field, our study compared an IL‐2‐Fc with full Fc effector function head‐to‐head against an ICK in the same solid tumor models to compare the relative toxicity and antitumor effects. One would expect that tumor‐targeted ICK would be less toxic and exhibit higher efficacy, but surprisingly, IL‐2‐Fc had less toxicity and equivalent antitumor efficacy compared to M5A‐IL‐2 ICK. We thus wanted to explore what mechanisms were responsible for these effects by analyzing immune cell populations in the tumor, spleen, and TDLNs. Tumor Treg depletion by IL‐2‐Fc was most evident in our early time point monotherapy studies, especially 1 day posttreatment in our MC38/CEA short term study, with some mice reaching a >50‐fold higher IFNγ^+^CD8^+^/Treg ratio (Figure 2K). The mice treated with ICK had a much milder increase that was maintained by the endpoint of the longer term MC38/CEA monotherapy study (Figure 1H). These differences lend clues as to how IL‐2‐Fc and ICK elicit their therapeutic effects. IL‐2‐Fc may have a more peripheral role in allowing cytotoxic immune cell activation and Treg depletion (note significant Treg depletion in the spleen not seen with ICK in both models Figures S1C and S3K), with a huge impact at the tumor in the short term. ICK may be acting more locally at the tumor with a primary role in CD8^+^ T cell activation that lasts long after injections (see Figure 1H; Figure S3H). To understand the inflammatory state of each myeloid subpopulation, functional assays would need to be performed. It has been shown that each tumor model has its own starting distribution of myeloid subpopulations, and It is important to keep in mind that the effects of IL‐2‐Fc and ICK on these starting subpopulations is also model and tissue dependent.^27^ However, the opposite shifts in monocyte and macrophage frequencies caused by IL‐2‐Fc and ICK in both models in the tumor in the short versus long term could be due to a recovery mechanism in the tumor at later time points given that IL‐2‐Fc or ICK monotherapies do not eradicate the tumor on their own. The pro‐inflammatory effects of SRT on tumor infiltrating myeloid cells described by others could be responsible for very efficient tumor eradication in our colon model when combined with either ICK or IL‐2‐Fc.^28^ This could be confirmed by future studies exploring the impact of SRT combination therapy on myeloid subpopulations in the tumor as well as blood and bone marrow assessment. Each of these differences and nuances between therapies and models could allow for a more informed perspective on dosing and timing once translated to the clinic.

Recently we have shown that an ICK combined with other modalities such as SRT is even more effective in tumor treatment than either modality alone.^13^ To extend development of this approach, we have now combined IL‐2‐Fc with SRT and compared it to ICK combined with SRT at the same doses and timing in the same animal model. It is well‐known that SRT may stimulate immune activation via adjuvants like cytokines or checkpoint inhibition post‐SRT.^29^ On the other hand, SRT combined with IL‐2 alone has shown high toxicity, including VLS, a toxicity that can be avoided with combined SRT plus ICKs.^29^ Aside from our M5A‐IL‐2 ICK SRT combination study mentioned previously,^13^ other examples include the NHS‐IL‐2 ICK combined with RT for metastatic non‐small cell lung cancer and L19‐IL‐2 ICK combined with RT for ED‐B+ cancers such as some colon and mammary carcinomas.^30^, ^31^ Each of these studies showed >70% complete tumor regression in preclinical tumor models expressing high target antigen, with high dependence on the presence of CD8^+^ T cells displayed in the L19‐IL‐2 ICK study.^13^, ^30^, ^31^ In contrast with tumor antigen targeting, another group demonstrated systemic immune activation contributing to the efficacy of combining hypofractionated radiation therapy with an IL‐2/anti‐IL‐2 complex.^32^ This IL‐2c agent was biased to bind the low affinity IL‐2 receptor on CD8^+^ T and NK cells, as opposed to our IL‐2 that targeted CD25 on Tregs with its natural affinity. Interestingly, this group found that their massive IL‐2c‐induced expansion of CD8^+^ T and NK cells was transient, yet they did not explore toxicity past immunoPET PD‐L1 tracer uptake seen in liver and lungs.^32^ Our IL‐2‐Fc construct may offer less toxicity than the IL‐2c agent via Treg depletion as opposed to hyper‐activation and expansion of CD8^+^ T and NK cells systemically. Further studies exploring types of radiation therapy, type of tumor, tumor location, as well as dosage and timing of IL‐2‐Fc will need to be explored, but our preliminary results show that this is a promising direction to take due to induction of immune memory.

Here we showed that human IL‐2‐Fc (K35E, C125S in human IL‐2; wild‐type IgG1) with IgG1 Fc effector function is equally efficacious as M5A‐IL‐2 ICK in immunocompetent CEA^+^ breast and colon cancer models. We showed that IL‐2‐Fc elicits immune memory when combined with SRT in our CEAtg colon cancer mouse model, despite not having the tumor‐specific targeting capability of M5A‐IL‐2 ICK. In contrast to M5A‐IL‐2, IL‐2‐Fc shows less toxicity in two tumor mouse models and elicits its antitumor effects primarily through Treg depletion. These properties make IL‐2‐Fc a versatile and unique antitumor agent that is worth pursuing for clinical use in multiple cancer indications.

## AUTHOR CONTRIBUTIONS

**Lindsay Williams:** Conceptualization (equal); data curation (equal); formal analysis (equal); investigation (lead); methodology (equal); project administration (lead); resources (equal); visualization (equal); writing – original draft (lead); writing – review and editing (lead). **Lin Li:** Formal analysis (equal); investigation (supporting); methodology (equal); writing – review and editing (equal). **Paul Yazaki:** Formal analysis (equal); methodology (equal); resources (equal); writing – review and editing (equal). **Patty Wong:** Data curation (equal); formal analysis (equal); investigation (supporting). **Teresa Hong:** Data curation (supporting); formal analysis (supporting); investigation (supporting). **Erasmus Poku:** Methodology (equal); resources (supporting); writing – review and editing (equal). **Susanta Hui:** Methodology (equal); resources (supporting). **Hemendra Ghimire:** Investigation (supporting). **John Shively:** Conceptualization (equal); data curation (equal); methodology (equal); project administration (equal); resources (equal); supervision (lead); writing – review and editing (equal). **Maciej Kujawski:** Conceptualization (equal); data curation (equal); methodology (equal); project administration (equal); resources (equal); supervision (lead); writing – review and editing (equal).

## FUNDING INFORMATION

This work was supported by the National Cancer Institute (P30CA033572).

## CONFLICT OF INTEREST STATEMENT

The authors declare no conflict of interest.

## Supporting information


Data S1.

## Data Availability

Raw data are available from the authors based on reasonable request.

## References

[1] Jenkins MK , Taylor PS , Norton SD , Urdahl KB . CD28 delivers a costimulatory signal involved in antigen‐specific IL‐2 production by human T cells. J Immunol. 1991;147(8):2461‐2466. doi:10.4049/jimmunol.147.8.2461 1717561

[2] Morgan DA , Ruscetti FW , Gallo R . Selective in vitro growth of T lymphocytes from Normal human bone marrows. Science. 1976;193(4257):1007‐1008. doi:10.1126/science.181845 181845

[3] Chinen T , Kannan AK , Levine AG , et al. An essential role for the IL‐2 receptor in T(reg) cell function. Nat Immunol. 2016;17(11):1322‐1333. doi:10.1038/ni.3540 27595233 PMC5071159

[4] Jiang T , Zhou C , Ren S . Role of IL‐2 in cancer immunotherapy. Onco Targets Ther. 2016;5(6):e1163462. doi:10.1080/2162402x.2016.1163462 PMC4938354 27471638

[5] Assier E , Jullien V , Lefort J , et al. NK cells and Polymorphonuclear neutrophils are both critical for IL‐2‐induced pulmonary vascular leak syndrome. J Immunol. 2004;172(12):7661‐7668. doi:10.4049/jimmunol.172.12.7661 15187148

[6] Gately MK , Anderson TD , Hayes TJ . Role of asialo‐GM1‐positive lymphoid cells in mediating the toxic effects of recombinant IL‐2 in mice. J Immunol. 1988;141(1):189‐200. doi:10.4049/jimmunol.141.1.189 3259967

[7] Peace DJ , Cheever MA . Toxicity and therapeutic efficacy of high‐dose interleukin 2. In vivo infusion of antibody to NK‐1.1 attenuates toxicity without compromising efficacy against murine leukemia. J Exp Med. 1989;169(1):161‐173. doi:10.1084/jem.169.1.161 2783332 PMC2189181

[8] Van Gool F , Molofsky AB , Morar MM , et al. Interleukin‐5–producing group 2 innate lymphoid cells control eosinophilia induced by interleukin‐2 therapy. Blood. 2014;124(24):3572‐3576. doi:10.1182/blood-2014-07-587493 25323825 PMC4256909

[9] Vazquez‐Lombardi R , Loetsch C , Zinkl D , et al. Potent antitumour activity of interleukin‐2‐fc fusion proteins requires fc‐mediated depletion of regulatory T‐cells. Nat Commun. 2017;8(1):15373. doi:10.1038/ncomms15373 28497796 PMC5437307

[10] Williams L , Li L , Yazaki PJ , et al. Generation of IL‐2‐Fc‐antibody conjugates by click chemistry. Biotechnol J. 2023;18(9):2300115. doi:10.1002/biot.202300115 37300381

[11] Rojas G , Carmenate T , Santo‐Tomás JF , et al. Directed evolution of super‐secreted variants from phage‐displayed human Interleukin‐2. Sci Rep. 2019;9(1):800. doi:10.1038/s41598-018-37280-5 30692603 PMC6349883

[12] Zheng X , Wu Y , Bi J , et al. The use of supercytokines, immunocytokines, engager cytokines, and other synthetic cytokines in immunotherapy. Cell Mol Immunol. 2022;19(2):192‐209. doi:10.1038/s41423-021-00786-6 35043005 PMC8803834

[13] Kujawski M , Sherman M , Hui S , et al. Potent immunomodulatory effects of an anti‐CEA‐IL‐2 immunocytokine on tumor therapy and effects of stereotactic radiation. Onco Targets Ther. 2020;9(1):1724052. doi:10.1080/2162402X.2020.1724052 PMC7028338 32117587

[14] Li L , Bading J , Yazaki PJ , et al. A versatile bifunctional chelate for radiolabeling humanized anti‐CEA antibody with in‐111 and Cu‐64 at either thiol or amino groups: PET imaging of CEA‐positive tumors with whole antibodies. Bioconjug Chem. 2008;19(1):89‐96. doi:10.1021/bc700161p 17988078 PMC2553277

[15] Nittka S , Krueger MA , Shively JE , et al. Radioimmunoimaging of liver metastases with PET using a 64Cu‐labeled CEA antibody in transgenic mice. PloS One. 2014;9(9):e106921. doi:10.1371/journal.pone.0106921 25226518 PMC4165898

[16] Cahan B , Leong L , Wagman L , et al. Phase I/II trial of Anticarcinoembryonic antigen Radioimmunotherapy, gemcitabine, and hepatic arterial infusion of Fluorodeoxyuridine Postresection of liver metastasis for colorectal carcinoma. Cancer Biother Radiopharm. 2017;32(7):258‐265. doi:10.1089/cbr.2017.2223 28910150 PMC5646801

[17] Wong JYC , Chu DZ , Williams LE , et al. A phase I trial of 90Y‐DOTA‐anti‐CEA chimeric T84.66 (cT84.66) radioimmunotherapy in patients with metastatic CEA‐producing malignancies. Cancer Biother Radiopharm. 2006;21(2):88‐100. doi:10.1089/cbr.2006.21.88 16706629

[18] Duffy MJ . Carcinoembryonic antigen as a marker for colorectal cancer: is it clinically useful? Clin Chem. 2001;47(4):624‐630. doi:10.1093/clinchem/47.4.624 11274010

[19] Goldstein MJ , Mitchell EP . Carcinoembryonic antigen in the staging and follow‐up of patients with colorectal cancer. Cancer Invest. 2005;23(4):338‐351. doi:10.1081/CNV-58878 16100946

[20] Lee JS , Park S , Park JM , Cho JH , Kim SI , Park BW . Elevated levels of preoperative CA 15‐3 and CEA serum levels have independently poor prognostic significance in breast cancer. Ann Oncol. 2013;24(5):1225‐1231. doi:10.1093/annonc/mds604 23230137

[21] Shao Y , Sun X , He Y , Liu C , Liu H . Elevated levels of serum tumor markers CEA and CA15‐3 are prognostic parameters for different molecular subtypes of breast cancer. PloS One. 2015;10(7):e0133830. doi:10.1371/journal.pone.0133830 26207909 PMC4514648

[22] Xu X , Clarke P , Gr S , et al. Targeting and therapy of carcinoembryonic antigen‐expressing tumors in transgenic mice with an antibody‐interleukin 2 fusion Protein1. Cancer Res. 2000;60(16):4475‐4484. 10969795

[23] Yazaki PJ , Sherman MA , Shively JE , et al. Humanization of the anti‐CEA T84.66 antibody based on crystal structure data. Protein Eng Des Sel. 2004;17(5):481‐489. doi:10.1093/protein/gzh056 15316127

[24] Clarke P , Mann J , Simpson JF , Rickard‐Dickson K , Primus FJ . Mice transgenic for human carcinoembryonic antigen as a model for immunotherapy. Cancer Res. 1998;58(7):1469‐1477. 9537250

[25] Kujawski M , Li L , Bhattacharya S , et al. Generation of dual specific bivalent BiTEs (dbBIspecific T‐cell engaging antibodies) for cellular immunotherapy. BMC Cancer. 2019;19(1):882. doi:10.1186/s12885-019-6056-8 31488104 PMC6727398

[26] Tripathi P , Tripathi P , Kashyap L , Singh V . The role of nitric oxide in inflammatory reactions. FEMS Immunol Med Microbiol. 2007;51(3):443‐452. doi:10.1111/j.1574-695X.2007.00329.x 17903207

[27] Zhang Y , Huang H , Coleman M , et al. VEGFR2 activity on myeloid cells mediates immune suppression in the tumor microenvironment. JCI Insight. 2021;6(23):e150735. doi:10.1172/jci.insight.150735 34673569 PMC8675197

[28] Gough MJ , Young K , Crittenden M . The impact of the myeloid response to radiation therapy. Clin Dev Immunol. 2013;2013:281958. doi:10.1155/2013/281958 23653658 PMC3638700

[29] Palata O , Hradilova Podzimkova N , Nedvedova E , et al. Radiotherapy in combination with cytokine treatment. Front Oncol. 2019;9:367. doi:10.3389/fonc.2019.00367 31179236 PMC6538686

[30] van den Heuvel MM , Verheij M , Boshuizen R , et al. NHS‐IL2 combined with radiotherapy: preclinical rationale and phase Ib trial results in metastatic non‐small cell lung cancer following first‐line chemotherapy. J Transl Med. 2015;13(1):32. doi:10.1186/s12967-015-0397-0 25622640 PMC4320467

[31] Zegers CM , Rekers NH , Quaden DH , et al. Radiotherapy combined with the Immunocytokine L19‐IL2 provides long‐lasting antitumor EffectsRadiotherapy in combination with the Immunocytokine L19‐IL2. Clin Cancer Res. 2015;21(5):1151‐1160. doi:10.1158/1078-0432.CCR-14-2676 25552483

[32] Jing H , Hettich M , Gaedicke S , Firat E , Bartholomä M , Niedermann G . Combination treatment with hypofractionated radiotherapy plus IL‐2/anti‐IL‐2 complexes and its theranostic evaluation. J Immunother Cancer. 2019;7(1):55. doi:10.1186/s40425-019-0537-9 30808414 PMC6390578

