# Supplementary material for: Comparison of IL‐2‐antibody to IL‐2‐Fc with or without stereotactic radiation therapy in CEA immunocompetent mice with CEA positive tumors
Source: Cancer Med. 2024 Feb 5;13(3):e6909. doi: 10.1002/cam4.6909 (PMC10905250; doi:10.1002/cam4.6909)
Supplement: Supplementary file 1 — Data S1. [file CAM4-13-e6909-s001.docx]

**­Comparison of IL-2-antibody to IL-2-Fc with or without stereotactic radiation therapy in CEA immunocompetent mice with CEA positive tumors**

Lindsay Williams^1^, Lin Li^1^, Paul J. Yazaki^1^, Patty Wong^1^, Teresa Hong^1^, Erasmus K. Poku^2^, Susanta Hui^3^, Hemendra Ghimire^3^, John E. Shively^1^, and Maciej Kujawski^1^

**SUPPLEMENTARY FIGURES**

**
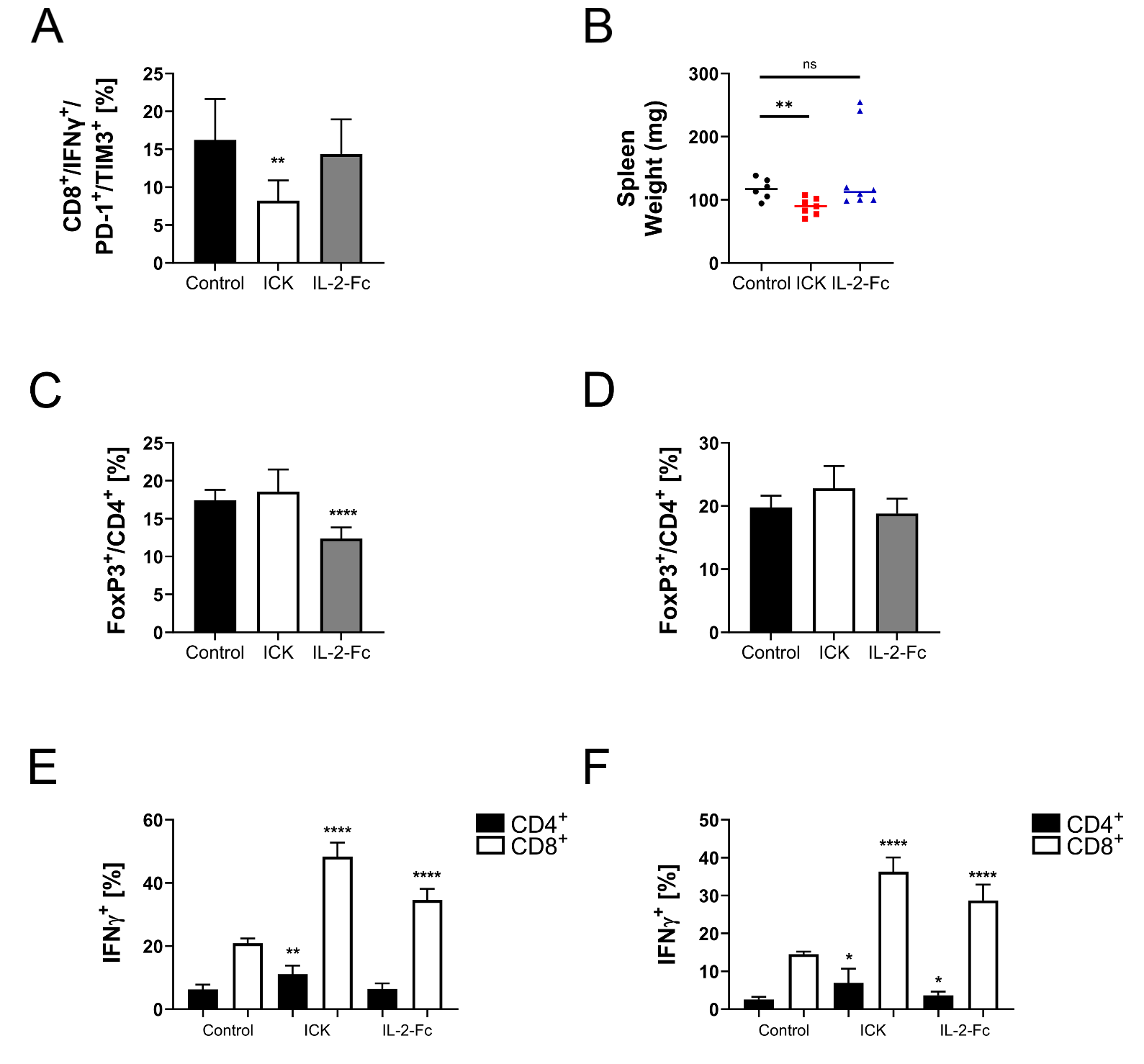
**

**Figure S1.** ICK and IL-2-Fc therapy in MC38/CEA subcutaneous CEAtg mouse model. **A.** Flow analysis of CD8^+^/IFNγ^+^/PD-1^+^/TIM3^+^ cells in tumors from **Figure 1**. **B.** Endpoint spleen mass from each experimental group (n = 6-8 per group). **C-F:** Flow analysis of cell frequencies at study endpoint (day 32) (n = 6-8 per group). **C.** FoxP3^+^/CD4^+^ (Treg) cells in spleen. **D.** FoxP3^+^/CD4^+^ (Treg) cells in tumor draining lymph node (TDLN). **E.** IFNγ^+^ CD4^+^ and CD8^+^ T cells in spleen. **F.** IFNγ^+^ CD4^+^ and CD8^+^ T cells in TDLN. ****p < .0001; ***p < .001; **p < .01; *p < .05; not significant (ns). (All statistics shown are in comparison to control values).


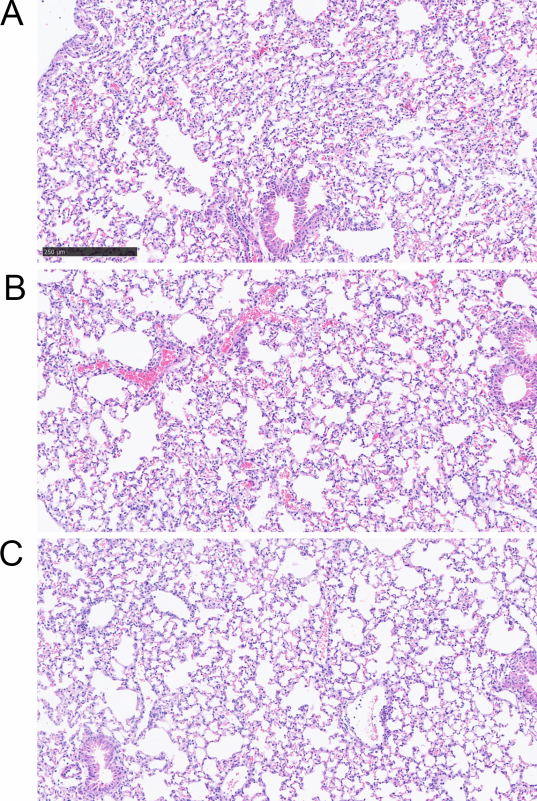


**Figure S2.** Histopathological analysis of lung from MC38/CEA bearing mice treated with ICK and IL-2-Fc. **A.** Representative H&E staining of endpoint lung section from control (untreated) untreated group. **B.** ICK treatment group. **C.** IL-2-Fc treatment group. **A-C:** scale bar = 250µm; all images at 10x magnification.


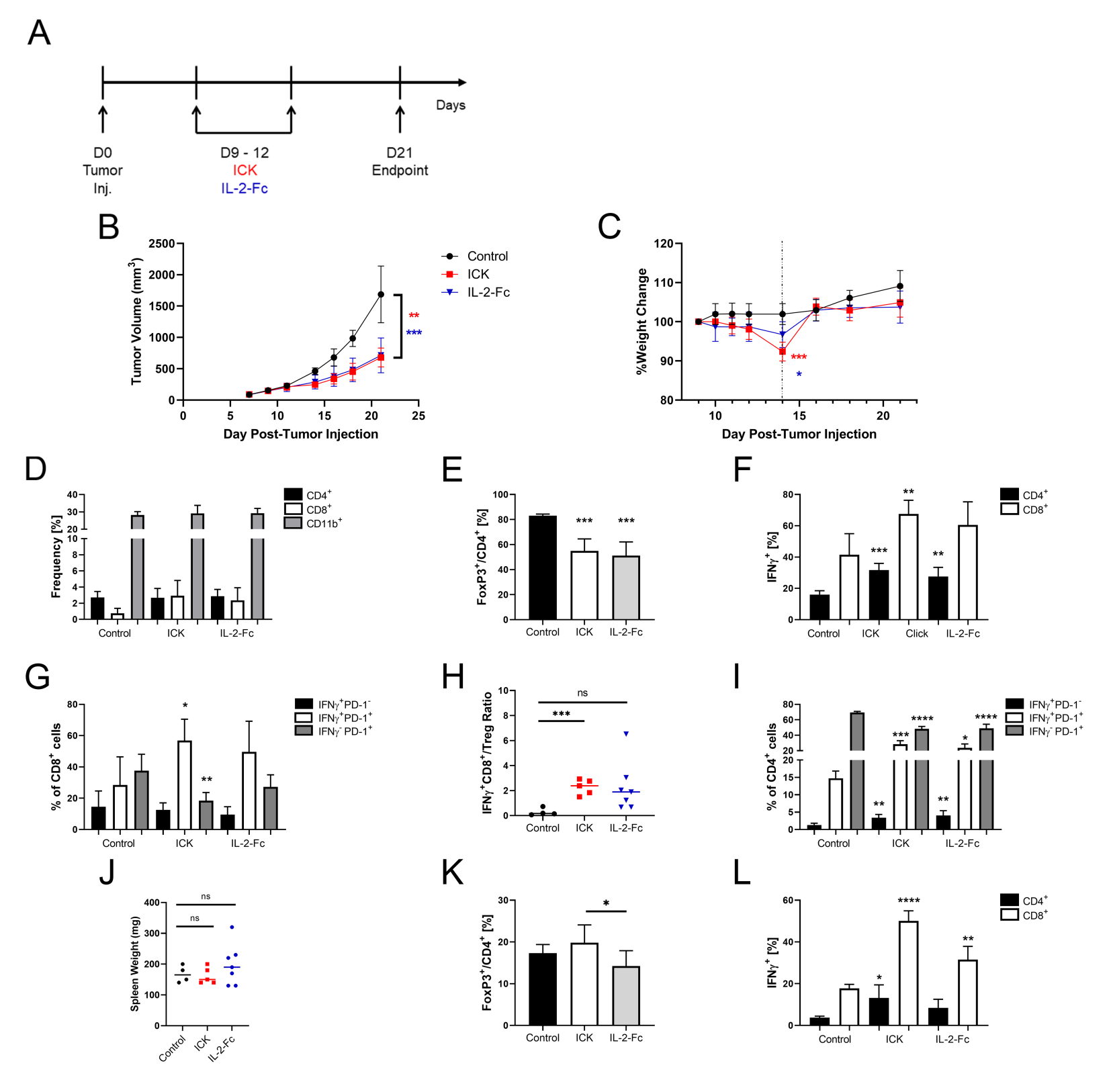


**Figure S3.** ICK and IL-2-Fc therapy in E0771/CEA orthotopic breast tumors in a CEAtg mouse model. **A.** Study design scheme. Orthotopic E0771/CEA (1 x 10^5^) tumor-bearing CEAtg mice were treated on days 9-12 after tumor implantation with intraperitoneal injection of 1 mg/kg ICK or molar equivalent of IL-2-Fc and euthanized on day 21. **B.** Tumor growth curves of control (untreated), ICK, and IL-2-Fc treatment groups (n = 5-7 per group). **C.** Percent weight change from baseline (100%). Dashed line represents time point chosen for statistical comparison (n = 5-7 per group). **D-I:** Flow analysis of cell frequencies from endpoint tumor digests (n = 4-7 per group). **D.** CD4^+^, CD8^+^, and CD11b^+^ leukocytes. **E.** FoxP3^+^/CD4^+^ (Tregs). **F.** IFNγ^+^ CD4^+^ and CD8^+^ T cells. **G.** IFNγ^+^PD-1^-^, IFNγ^+^PD-1^+^, and IFNγ^-^PD-1^+^ CD8^+^ T cells. **H.** IFNγ^+^CD8^+^/Treg ratio (out of all live cells in tumor). **I.** IFNγ^+^PD-1^-^, IFNγ^+^PD-1^+^, and IFNγ^-^PD-1^+^ CD4^+^ T cells. **J.** Endpoint spleen weight from each experimental group. (n = 4-7 per group). **K.** FoxP3^+^/CD4^+^ (Treg) cells in spleen. **L.** IFNγ^+^ CD4^+^ and CD8^+^ T cells in spleen. ****p < .0001; ***p < .001; **p < .01; *p < .05. (All statistics shown are in comparison to control values unless explicitly shown).


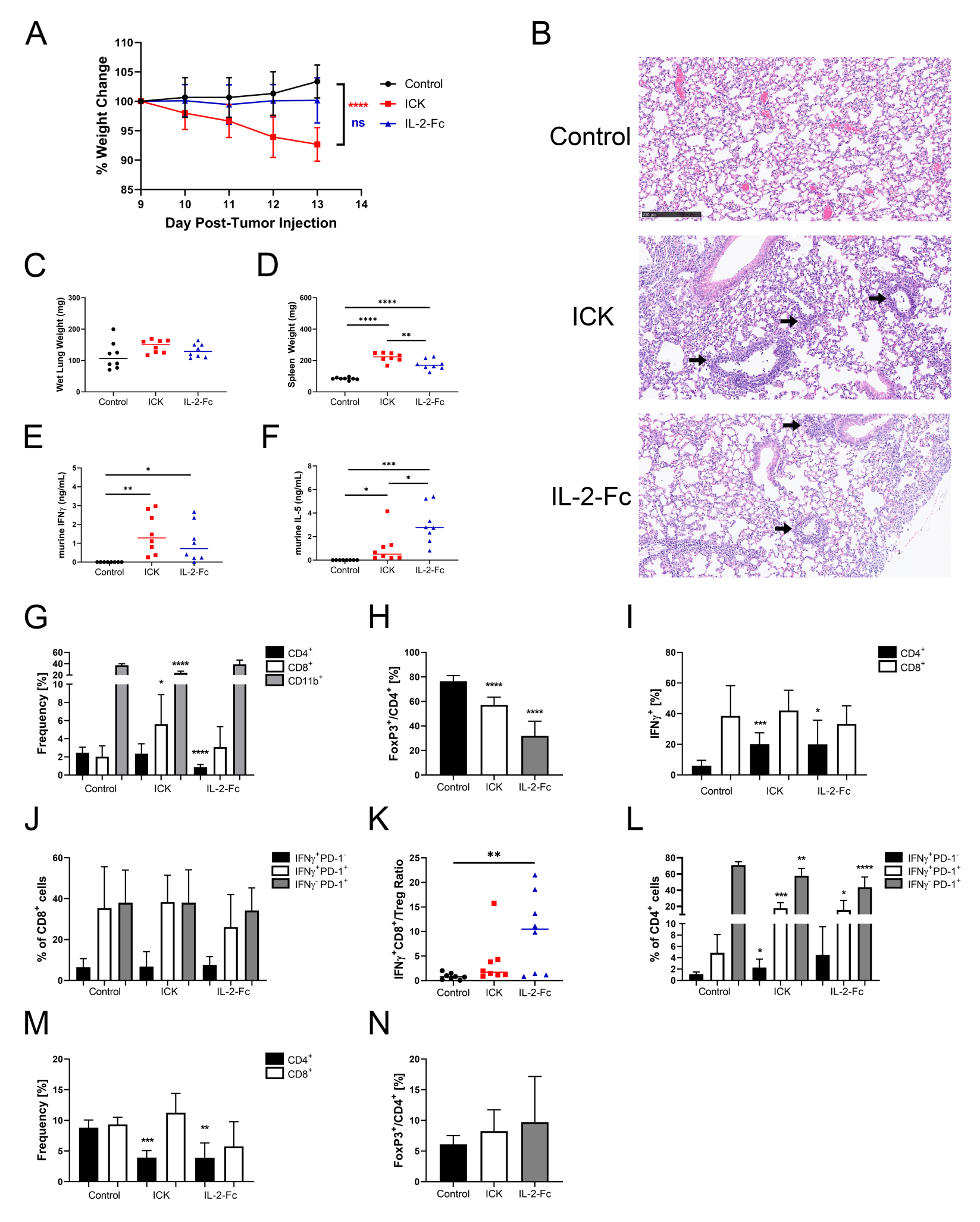


**Figure S4.** Comparison of ICK and IL-2-Fc toxicity in orthotopic E0771/CEA mouse model. Mice processed one day post-treatment cessation following scheme in **Figure S3A**. n = 5-8 (pooled) for all graphs. **A.** Percent weight change from baseline (100%). **B.** Representative H&E staining of endpoint lung section from control (untreated), ICK, and IL-2-Fc experimental groups (scale bar = 250µm; all images at 10x magnification; black arrow indicates areas of increased cellularity near blood vessels). **C.** Wet lung weight at endpoint (day 13). **D.** Spleen weight at endpoint (day 13). **E.** Plasma IFNγ concentration at endpoint. **F.** Plasma IL-5 concentration at endpoint. **G-N:** Flow analysis of cell frequencies from endpoint samples. **G.** CD4^+^, CD8^+^, and CD11b^+^ leukocytes in the tumor. **H.** FoxP3^+^/CD4^+^ (Treg) cells in the tumor. **I.** IFNγ^+^ CD4^+^ and CD8^+^ T cells in the tumor. **J.** IFNγ^+^PD-1^-^, IFNγ^+^PD-1^+^, and IFNγ^-^PD-1^+^ CD8^+^ T cells in the tumor. **K.** IFNγ^+^CD8^+^/Treg ratio of all live cells in tumor. **L.** IFNγ^+^PD-1^-^, IFNγ^+^PD-1^+^, and IFNγ^-^PD-1^+^ CD4^+^ T cells in the tumor. **M.** CD4^+^ and CD8^+^ T cells in the blood. **N.** FoxP3^+^/CD4^+^ (Treg) cells in the blood. ****p < .0001; ***p < .001; **p < .01; *p < .05. (All statistics shown are in comparison to control values unless explicitly shown).


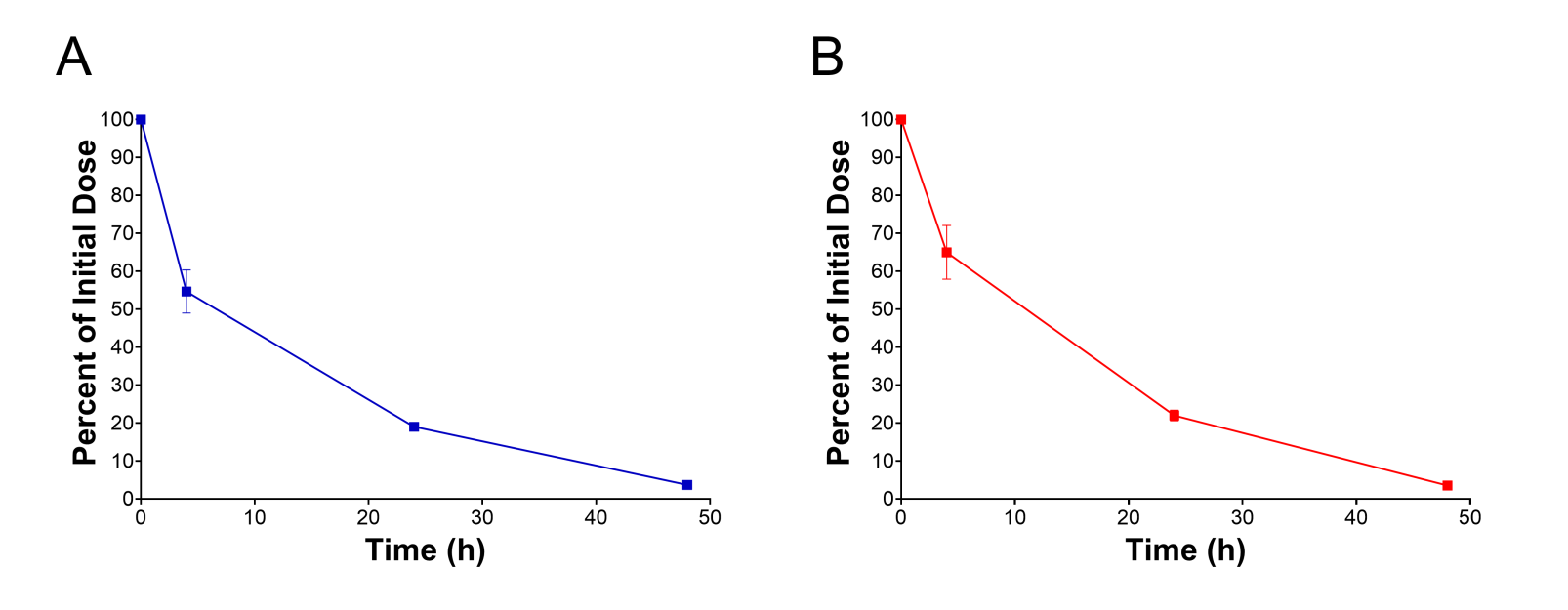


**Figure S5.** PK of IL-2-Fc and M5A-IL-2 ICK. **A.** PK of ^64^Cu-DOTA-IL-2-Fc. (n = 3). **B.** PK of ^64^Cu-DOTA-ICK. (n = 2). (PK taken from CEAtg mice with tumors as in **Figure 5**). Abbreviations: Hour (h), pharmacokinetics (PK).
